# High-Throughput Sequencing Profiles About lncRNAs and mRNAs of Ovarian Granulosa Cells in Polycystic Ovary Syndrome

**DOI:** 10.3389/fmed.2021.741803

**Published:** 2021-11-22

**Authors:** Yanjun Zheng, Yuehong Bian, Richao Wu, Wei Chen, Linlin Fu, Ping Li, Ying Wang, Xiao Yang, Shigang Zhao, Yuhua Shi

**Affiliations:** ^1^Center for Reproductive Medicine, Cheeloo College of Medicine, Shandong University, Jinan, China; ^2^Key Laboratory of Reproductive Endocrinology of Ministry of Education, Shandong University, Jinan, China; ^3^Shandong Key Laboratory of Reproductive Medicine, Jinan, China; ^4^Shandong Provincial Clinical Research Center for Reproductive Health, Jinan, China; ^5^National Research Center for Assisted Reproductive Technology and Reproductive Genetics, Shandong University, Jinan, China

**Keywords:** ovarian granulosa cells, lncRNA, mRNA, pcos, high-throughput sequencing

## Abstract

Polycystic ovary syndrome (PCOS) is one of the most common endocrine disorders in women of reproductive age, which is characterized by ovulatory dysfunction, clinical and/or biochemical androgen excess, polycystic ovaries on ultrasound and genetic heterogeneity. It was well-accepted that many lncRNAs and mRNAs were associated with PCOS, however, remain unclear. Therefore, the purpose of our study was to examine different expression profiles of lncRNAs and mRNAs in ovarian granulosa cells (GCs) in PCOS and Controls, and identify the correlation between lncRNAs, mRNAs and clinical parameters. Sixty five PCOS patients and 65 Controls were enrolled in this study and adopted standard long agonist protocols or GnRH antagonist protocols. Then 6 GCs samples in each group were subjected to high-thoughput sequencing and the remaining samples were used for the further verification by quantitative real-time PCR (qRT-PCR). Gene Oncology (GO), Kyoto Encyclopedia Genes and Genomes (KEGG) enrichment analysis were performed. We predicted the relationship between lncRNAs and mRNAs by Cytoscape software. According to the expression level of lncRNAs, mRNAs and the clinical parameters, we also explored their relationship and evaluate their predictive values for embryos quality and PCOS. We identified 1,049 differential expressed lncRNAs and 3,246 mRNAs (fold-change ≥2, *p*-value < 0.05). Seven lncRNAs (NONHSAT101926.2, NONHSAT136825.2, NONHSAT227177.1, NONHSAT010538.2, NONHSAT191377.1, NONHSAT230904.1, ENST00000607307) and 3 mRNAs (EREG, ENTPD6, YAP1) were validated consistent with sequence profile. Seven lncRNAs were related to hormone level and follicle counts, 3 mRNAs had connections with lipid metabolism. The area under curve (AUC) of 7 lncRNAs were valuable in distinguishing patients with PCOS from Controls. The AUC of NONHSAT230904.1 and NONHSAT227177.1 were 0.6807 and 0.6410, respectively, for distinguishing whether the rate of high-quality embryos exceeds 50%. Our study showed that the GCs lncRNAs and mRNAs were involved in the occurrence and development of PCOS, which contribute to clarify the pathogenesis mechanism of PCOS.

## Introduction

Polycystic ovary syndrome (PCOS) is a common endocrine disorder affecting 8–13% of women of reproductive age (1). It is primarily characterized by ovulatory dysfunction, clinical and/or biochemical androgen excess, polycystic ovaries on ultrasound, and genetic heterogeneity (2). There are many other symptoms or signs that accompany PCOS, such as hyperandrogenemia, sleep apnea, and metabolic problems including insulin resistance, diabetes, acanthosis nigricans, and non-alcoholic fatty liver (3). In addition, certain psychological problems can also arise in such cases (4). It is now believed that environmental and genetic factors cause PCOS (5), but its exact etiology remains unclear.

Long non-coding RNAs (lncRNAs) are a type of non-coding RNA with a length exceeding 200 nucleotides. They contribute to transcriptional and post-transcriptional regulation, and are broadly classified as signaling molecules, decoy molecules, guide molecules, or scaffold molecules (6). LncRNAs, as “transcriptional noise,” have relatively low expression levels compared with mRNAs and lack protein-coding capacity (7). In recent years, many studies have revealed that lncRNAs exhibit certain characteristics of mRNAs. For instance, lncRNAs are transcribed by RNA polymerase II, equipped with a 3′ poly (A) tail and a 5′ cap, and contain a promoter and multiple exons (8, 9). These features enable lncRNAs to interact with DNA, RNA, and proteins to directly regulate several biological processes, including chromatin modification, RNA transcription, pre-mRNA splicing, mRNA translation, and gene expression (10, 11). In addition, some studies have been found that lncRNAs have target-mimetic, sponge/decoy functions on microRNAs (12). Many studies also have revealed the roles of microRNA in various diseases (13, 14). At the same time, several studies have shown a wide range of interactions among mRNAs, microRNAs, and lncRNAs (15).

Several studies on lncRNAs have described the association with PCOS. The results of lncRNAs profiles in cumulus cells revealed that lncRNAs may contribute to the occurrence of PCOS and affect oocyte development (16). In addition, Liu et al. reported that lncRNAs may play a role in the proliferation and steroidogenesis of human granulosa cells (GCs) (17). The profiles of lncRNAs in GCs from women with PCOS with or without hyperandrogenism showed that lncRNAs may play key roles in steroidogenesis and metabolism (18). The lncRNA PVT1 regulates the secretion of estradiol (E_2_) and progesterone (P_4_), and the proliferation and apoptosis of GCs in PCOS *via* the lncRNA PVT1/microRNA-17-5p/PTEN axis (19). By constructing gene co-expression networks, it was revealed that there were many co-regulatory relationships among lncRNAs, mRNAs, and PCOS phenotypes from lncRNAs and mRNAs profiles in follicular fluid from mature and immature ovarian follicles (6). Furthermore, lncRNAs may have a certain impact on embryo quality. It was reported in the literature that lncRNAs could influence zebra embryonic development by regulating the Yax gene (20). However, so far, the mechanism of lncRNAs affect embryo quality is not very clear.

In addition, lncRNAs also played an important role in lipid metabolism. Low level of plasm high-density lipoprotein cholesterol (HDL-C) was a risk factor in cardiovascular (21). Xiao-Hua Yu et al. found that lncRNA kcnq1ot1 overexpression markedly decreased plasma HDL-C level, and kcnq1ot1 could promote lipid accumulation and accelerate the development of atherosclerosis through the miR-452-3p/HDAC3/ABCA1 pathway (22). Moreover, Xinping Wang et al. performed microarray analysis of long non-coding RNA expression profiles in low high-density lipoprotein cholesterol disease and found that certain lncRNAs could cause low high-density lipoprotein cholesterol disease (23). LncRNAs could modulate the transfer of lipid molecules. Apolipoprotein A1 (APOA1) is the primary protein in HDL complexes, which are in charge of the transport of excess cholesterol to the liver. The lncRNA APOA1-AS was observed negatively regulated APO A1 transcription in both *in vivo* and *in vitro* liver models (24).

Therefore, to explore the potential pathogenetic role of lncRNAs in patients with PCOS, we also carried out high-throughput sequencing of mRNAs expression profiles to conduct a network including mRNAs, lncRNAs and possible microRNAs.

However, the regulatory mechanisms and biological functions of lncRNAs in PCOS are still not fully understood. Hence, the aim of this study is to investigate the differences of lncRNA and mRNAs profiles between PCOS and Controls, and to discover novel prognostic markers and therapeutic targets of this condition.

## Materials and Methods

### Participants and Group Criteria

Ovarian GCs were collected from 65 PCOS patients (PCOS group) and 65 patients with an indication of male factor infertility serving as Controls (Control group), all of whom underwent *in vitro* fertilization (IVF) or intracytoplasmic sperm injection (ICSI) at the Center for Reproductive Medicine, Shandong University, between December 2018 and December 2019. These patients agreed to undergo long protocol and/or GnRH antagonist protocol to promote follicle development (25, 26). The PCOS patients were diagnosed based on Rotterdam revised criteria (27, 28) after excluding patients with Cushing's syndrome, congenital adrenal hyperplasia and androgen-secreting tumors. The exclusion criteria of the two groups were age ≥40 years old; body mass index (BMI) ≥30 kg/m^2^; basal follicle stimulating hormone (FSH) level >12 mIU/L; and systemic diseases, endometriosis, abnormal prolactin levels or thyroid function, immune diseases, recurrent abortion, abnormal chromosomal. This study was approved by the ethics committee of the Reproductive Hospital Affiliated to Shandong University. Written informed consent was obtained from each patient.

### Clinical and Endocrine Parameters of PCOS Patients and Controls Who Were Subjected to High-Throughput Sequencing

We selected 6 GCs of PCOS patients and 6 GCs of Controls subjected to high-throughput sequencing. The clinical characteristics of the PCOS patients and Controls are shown in Table 1, including age, body mass index (BMI, kg/m^2^), fasting plasma glucose (FPG), plasma luteinizing hormone (LH), plasma follicle-stimulating hormone (FSH), plasma free testosterone (T), anti-Müllerian hormone (AMH). In addition, the antral follicle count (AFC) for follicles with a diameter of 2–9 mm was determined by transvaginal ultrasound. It should be note that increasing BMI appears to be causal for PCOS (29), and PCOS patients with high BMI could be frequently observed in clinical practice. For this reason, the difference in BMI found between PCOS and control groups was not taken into account.

**Table 1:** Clinical and endocrine parameter of PCOS patients and controls enrolled in high throughput sequencing.

**Basic parameters**	**PCOS (***n*** = 6)**	**Control (***n*** = 6)**	***p*** **-value**
Age (years)	28.83 ± 2.98	28.67 ± 2.66	0.9488
BMI (kg/m2)	25.82 ± 2.78	21.06 ± 1.42	0.0039
FBG (mmol/L)	5.24 ± 0.33	4.93 ± 0.62	0.2997
LH (IU/L)	7.36 ± 2.49	5.46 ± 1.63	0.1488
FSH (IU/L)	5.39 ± 1.25	6.63 ± 1.78	0.1944
T (ng/dL)	31.43 ± 22.48	24.33 ± 15.76	0.5404
AMH (ng/ml)	8.15 ± 2.98	5.07 (4.132)	0.1453

*FBG, fasting blood glucose; LH, luteinizing hormone; T, testosterone; AMH, anti-mullerian hormone*.

### Follicular Fluid Collection and Retrieval of Ovarian Granulosa Cells

The two groups underwent controlled ovarian stimulation (COS) as described above. All patients were administered chorionic gonadotropin (hCG) at a dose of 4,000–8,000 IU was intramuscularly injected when at least two dominant follicles reached 18–20 mm in diameter. Oocytes were retrieved 36 h after hCG injection, while GCs were collected from the follicular fluid without blood contamination (30). GCs were purified by magnetic-activated cell sorting (Miltenyi Biotec, Bergisch Gladbach, Germany), in accordance with the manufacturer's instructions. GCs and follicular fluid samples were stored at −80°C. Total RNA was extracted from GCs using TRIzol Reagent (Life Technologies, Shanghai, China), in accordance with the manufacturer's instructions.

### Library Construction for RNA-Seq and Sequencing Procedures

To assess the total RNA, Agilent Bioanalyzer 2,100 (Agilent Technologies, CA, USA) was used to evaluate the RNA integrity number (RIN). Qubit^®^3.0 Fluorometer (Thermo Fisher, USA) was used to assess total RNA levels and purity. We used the TruSeq^®^Stranded Total Sample Preparation kit (Illumina, USA) to prepare the strand-specific libraries strictly following the manufacturer's instructions. Purified libraries were quantified using Qubit^®^ 2.0 Fluorometer (Life Technologies, USA) and confirmed using Agilent 2,100 Bioanalyzer (Agilent Technologies, USA) to confirm the insert size and calculate the molar concentration. Then, cluster was generated by cBot with the library diluted to 10 pM, followed by sequencing on HiSeq X Ten (Illumina, USA). The library construction and sequencing were performed at Shanghai Sinomics Corporation.

### LncRNA Microarray Analysis

GCs selected for microarray analysis were collected from 6 PCOS patients and 6 Controls. To preprocess the sequenced raw reads, we filtered out rRNA reads, sequencing adapters, short-fragment reads, and other low-quality reads. We mapped the cleaned reads to the human GRCh 38 reference genome using Tophat v2.0.9, allowing two mismatches. After genome mapping, Cufflinks v2.1.1 was run with a reference annotation to generate FPKM values for known gene models, and FPKM values refer to fragments per kilobase of exon model per million mapped fragments. We used Cuffdiff to identify these differentially expressed genes and set the *p*-value significance threshold in multiple tests. According to the FPKM of each sample, the fold changes were estimated. Finally, hierarchical clustering was performed to display the genes with distinct expression patterns among the samples. All raw sequencing data have been submitted to the Gene Expression Omnibus (GEO) database (https://www.ncbi.nlm.nih.gov/geo/).

### Pathway Analysis

Gene Ontology (GO; www.geneontology.org/) analysis was mainly used to analyze the main function of putative target genes. And Kyoto Encyclopedia Genes and Genomes (KEGG; www.genome.jp/kegg) analysis was performed to identify molecular pathways that were potentially altered.

### Quantitative Real-Time PCR (qRT-PCR)

To confirm the microarray results, we chose some lncRNAs and mRNAs to test their expression in more GCs samples, in line with the screening criteria, including |fold-change| ≥2, *p*-value < 0.05, sequence length <3,000 bp, and count >10. We used about 30 PCOS patients and 30 Control patients to confirm the expression trends of every lncRNAs and mRNAs that we chose. Due to the limited size of RNA and cDNA from every GCs, a total of 59 PCOS GCs and 59 Control GCs were enrolled, which were enough to validate these lncRNAs and mRNAs we screened. Total RNA was extracted from GCs using TRIzol Reagent (Life Technologies, Shanghai, China) and reversed-transcribed into cDNA using the PrimeScript™ RT Reagent Kit with gDNA Eraser (Takara, China), in accordance with the manufacturer's instructions. We quantified the levels of lncRNAs and mRNAs that we chose by qRT-PCR on a LightCycler 480 system, in accordance with the manufacturer's instructions with TB Green™ Premix Ex Taq™ II (Takara, China). We normalized the levels of lncRNAs and mRNAs using GAPDH expression. Finally, we calculated the relative expression level of each lncRNA by the 2^−ΔΔCT^ method. The clinical and endocrine parameters of the PCOS group and Control group are presented in Table 2, while the primers used for qRT-PCR are presented in Table 3.

**Table 2:** Clinical and endocrine parameter of PCOS patients and controls for qRT-PCR.

**Basic parameters**	**PCOS (***n*** = 59)**	**Control (***n*** = 59)**	***p*** **-value**
Age (years)	29.32 ± 4.07	30.63 ± 3.01	0.0501
BMI (kg/m2)	26.47 ± 3.39	21.78 ± 2.60	<0.0001
FBG (mmol/L)	5.39 ± 0.39	5.20 ± 0.43	0.02
LH (IU/L)	9.93 (7.80)	4.83 ± 1.64	<0.0001
FSH (IU/L)	5.64 ± 1.45	6.13 ± 1.72	0.1020
T (ng/dL)	38.62 ± 17.79	20.75 ± 8.38	<0.0001
AMH (ng/ml)	8.82 ± 4.05	4.34 (3.66)	<0.0001

*Note: FBG, fasting blood glucose; LH, luteinizing hormone; T, testosterone; AMH, anti-mullerian hormone*.

**Table 3:** Primer sequences for qRT-PCR.

	**Primer sequences**
GAPDH	F: 5'GCACCGTCAAGGCTGAGAAC3'
	R: 3'TGGTGAAGACGCCAGTGGA5'
NONHSAT101926.2	F: 5' GAGCGAGTGAACCAAGAAGAG 3'
	R: 5' GCCTGCTGGATTATGAACAAC 3'
NONHSAT230904.1	F: 5' AGGTTGTGGTTATTGGTGCTG 3'
	R: 5' ATTGTTGCTTCTGGCTTTCTC 3'
NONHSAT136825.2	F: 5' CAACCAATCAGCAGGACCATT 3'
	R: 5' CCCACCTCAGACTCCCAAAGT 3'
NONHSAT010538.2	F: 5' CAACCAATCAGCAGGACCATT 3'
	R: 5' TCTTGACTCCATCCACCATAA 3'
NONHSAT227177.1	F: 5' CGAAACTCACCGAAGGAAACA 3'
	R: 5' ACCGCTTACGCATCTAACCAA 3'
NONHSAT191377.1	F: 5' GTGGGCTTCTACTTATTTCTTG 3'
	R: 5' ACGCCATCTGTTGGAGTCATC 3'
ENST00000607307	F: 5' CATCACCACTCCCTAATCTCA 3'
	R: 5' CTTGGACAATACCTGGCTTTC 3'
ENTPD6	F: 5'AAGTACGTGTGTCGGACCCT3'
	R: 3'GACGTAGGTGAGGTCCATGC5'
EREG	F: 5'ACGTGTGGCTCAAGTGTCAA3'
	R: 3'AGTGTTCACATCGGACACCA5'
YAP1	F: 5'GCGGCTGAAACAGCAAGAAC3'
	R: 3'TTGGTAACTGGCTACGCAGG5'

*qRT-PCR, quantitative real-time polymerase chain reaction*.

### Predict the Relationship Between LncRNAs and MRNAs After Verification

We used Cytoscape software to predict the relationship between lncRNAs and mRNAs which have been verified by expanding number of samples in each group. By integrating the information of the lncRNAs and the relationship between the mRNAs, we could obtain a regulatory network diagram between the lncRNAs and mRNAs.

### Statistical Analysis

We used SPSS 26.0 (SPSS, Chicago, IL, USA), GraphPad Prism 8.0 (GraphPad Software, CA, USA), and R studio for the statistical analyses. Cytoscape was used to construct a lncRNA–microRNA-mRNA interaction network. Data are reported as mean ± standard deviation. Student's *t*-test was used for between-group comparisons, parameters not normally distributed were presented as median and compared by non-parametric test. The Pearson's correlation analysis was used for analyzing linear associations. Receiver operating characteristic (ROC) curve analysis was used for assessing the efficiency of lncRNAs in distinguishing PCOS samples from Controls and different quality of embryos. A *p*-value of < 0.05 was regarded as indicating statistical significance.

## Results

### Expression Profiles of LncRNAs and MRNAs in PCOS Patients and Controls

**Figure 1:**
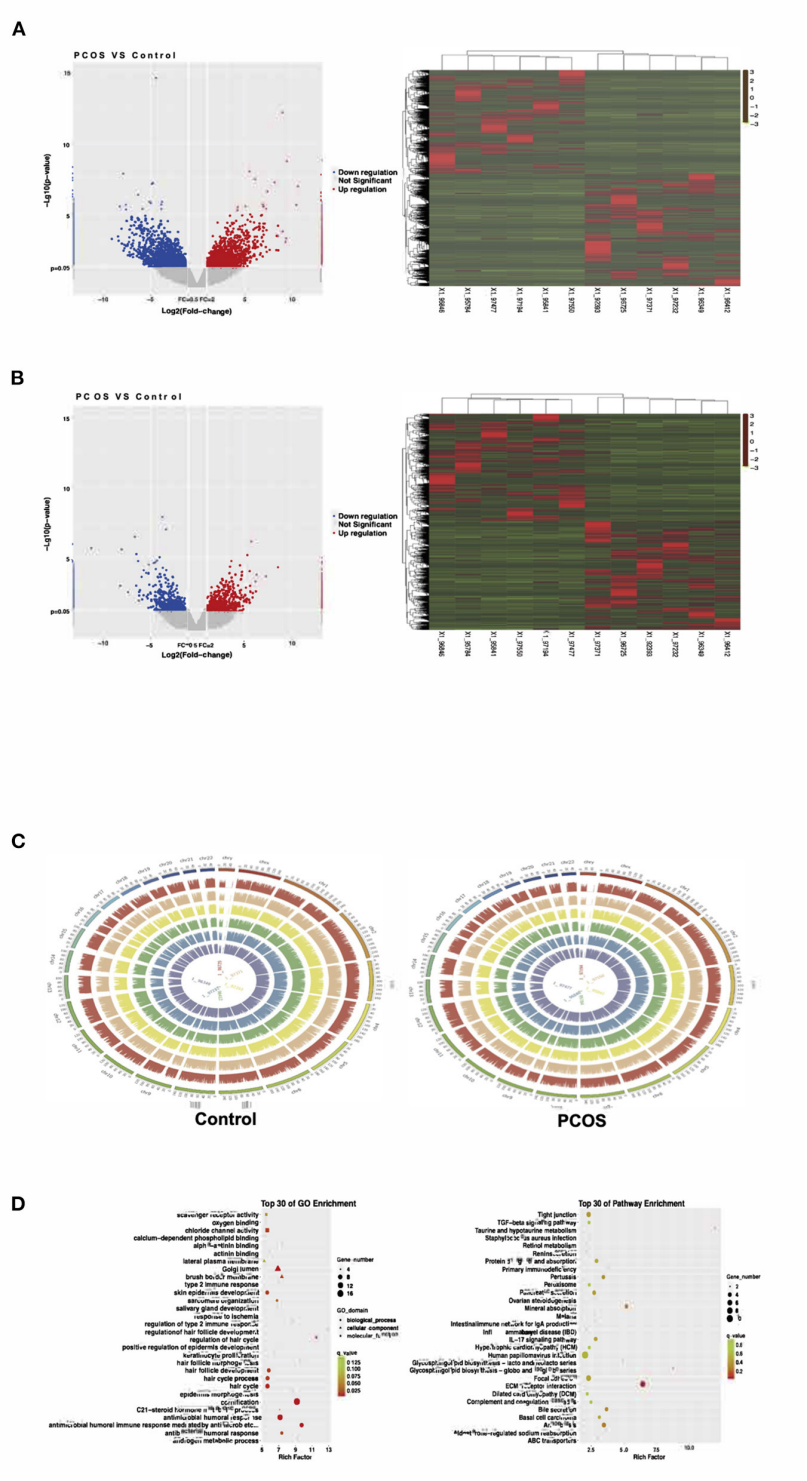
The expression of ovarian granulosa cells mRNAs and lncRNAs from polycystic ovary syndrome (PCOS) patients and controls. **(A)** Volcano map and Heat map of differentially expressed mRNAs (*p* < 0.05). **(B)** Volcano map and Heat map of differentially expressed lncRNAs (*p* < 0.05). **(C)** The distribution of differentially expressed lncRNAs in human chromosomes. **(D)** Gene Ontology (GO) and Kyoto Encyclopedia of Genes and Genomes (KEGG) pathway analysis for all target genes.

In our study, 54,615 lncRNAs and 123,141 mRNAs were detected in six PCOS patients and six Control patients by the high-throughput sequencing of lncRNAs. A Volcano plot was used to present the *p*-values and fold-changes of lncRNAs and mRNAs that were differentially expressed between the two groups. A heatmap was used to show the expression profiles of lncRNAs and mRNAs in PCOS patients and Controls. To obtain more detailed results, hierarchical clustering analysis was used to reveal the distribution of each of the lncRNAs and mRNAs (Figures 1A,B).

Compared with the levels in the Controls, the expression of 1,049 lncRNAs differed (|fold-change| ≥2, *p*-value < 0.05), of which 517 were upregulated and 532 downregulated. According to the same screening criteria, 3,246 mRNAs presented differential expression, with 1,551 upregulated and 1,695 downregulated in PCOS. Tables 4, 5 showed the top 10 most upregulated and downregulated lncRNAs and mRNAs according to the fold changes and *p*-values. The distribution of differentially expressed lncRNAs in the human chromosomes is illustrated in Figure 1C. These differentially expressed lncRNAs mainly derived from Chr.1 (10.20%, 107/1049), followed by Chr.2 (7.24%, 76/1049) and Chr.17 (6.39%, 67/1049). It should be note that the X and Y chromosome in human have both homologous and non-homologous part, and the alleles only present on homologous chromosome part, which lead to a small part of alleles were aligned to the Y chromosome.

**Table 4:** The ten most upregulated and ten most downregulated lncRNAs.

**LncRNA ID**	**Locus**	**Length**	**log2FC**	***p*** **-value**	**Up down**
NONHSAT101926.2	5:69494213-69710514	1,334	5.6812934	7.23E-07	UP
NONHSAT228733.1	10:4754001-4764085	8,653	5.3775428	5.08E-06	UP
NONHSAT169047.1	14:20457859-20460670	1,217	3.6807986	1.31E-05	UP
ENST00000622160	16:525155-527407	2,253	4.6199019	2.66E-05	UP
ENST00000565241	15:24558217-24568121	480	3.5762505	3.49E-05	UP
NONHSAT188617.1	20:25222488-25226711	1,823	8.5959584	3.69E-05	UP
NONHSAT248142.1	4:9034510-9073155	1,379	5.6142536	6.36E-05	UP
NONHSAT241372.1	2:207239531-207253199	4,902	4.7975169	7.56E-05	UP
NONHSAT010539.2	1:241413750-241433910	829	3.4763996	9.24E-05	UP
NONHSAT198617.1	4:55175478-55188459	11,577	4.1151228	0.0001212	UP
NONHSAT249144.1	5:56124101-56131804	3,350	−3.747726	1.33E-08	DOWN
NONHSAT258791.1	X:74249780-74250942	1,163	−3.343363	1.01E-07	DOWN
NONHSAT253044.1	7:1160462-1172551	10,865	−6.661202	3.35E-07	DOWN
NONHSAT036748.2	14:49853618-49853934	317	−11.25729	2.12E-06	DOWN
NONHSAT175809.1	17:1344551-1354292	1,078	−8.029133	2.74E-06	DOWN
NONHSAT250224.1	5:91301045-91314402	4,738	−6.368435	4.38E-06	DOWN
NONHSAT147588.2	7:100318828-100335913	2,663	−3.895616	6.75E-06	DOWN
NONHSAT028687.2	12:55829608-55836246	3,070	−6.206145	1.23E-05	DOWN
NONHSAT171425.1	15:34084027-34090460	685	−4.301474	1.65E-05	DOWN
NONHSAT092802.2	3:154254186-154259610	1,970	−4.460229	2.17E-05	DOWN

*Fold-change (FC) is presented as lncRNAs expression in women with vs, without polycystic ovary syndrome (PCOS). Up and Down are presented as the expression trend of lncRNAs in in women with vs. without polycystic ovary syndrome (PCOS)*.

**Table 5:** The ten most upregulated and ten most downregulated mRNAs.

**Transcript id**	**Gene id**	**Gene name**	**Locus**	**Length**	**Log2FC**	***p*** **-value**	**Up down**
ENST00000489456	ENSG00000123999	INHA	2:219569162-219575053	645	8.971234	6.33E-13	UP
ENST00000487182	ENSG00000124593	AL365205.1	6:41780772-41789891	3,043	9.4290867	1.90E-09	UP
ENST00000621410	ENSG00000274349	ZNF658	9:66900729-66921439	4,025	5.5012977	9.71E-09	UP
ENST00000340800	ENSG00000068366	ACSL4	X:109641337-109733392	5,333	6.0789287	3.52E-08	UP
ENST00000545675	ENSG00000086475	SEPHS1	10:13317439-13348298	2,988	8.1664593	6.00E-08	UP
ENST00000533455	ENSG00000164733	CTSB	8:11844355-11868106	2,009	10.607742	1.14E-07	UP
ENST00000370007	ENSG00000166197	NOLC1	10:102152415-102163870	3,727	7.3838865	2.44E-07	UP
ENST00000431736	ENSG00000110344	UBE4A	11:118359644-118399207	6,061	6.9222346	2.18E-06	UP
ENST00000439383	ENSG00000175166	PSMD2	3:184300581-184309048	2,633	10.452645	2.32E-06	UP
ENST00000591601	ENSG00000099875	MKNK2	19:2037465-2050887	3,582	5.072788	2.97E-06	UP
ENST00000248151	ENSG00000127589	TUBBP1	8:30352008-30353342	1,335	−4.411747	2.23E-15	DOWN
ENST00000495891	ENSG00000163659	TIPARP	3:156681166-156706750	2,700	−7.881976	1.39E-08	DOWN
ENST00000367256	ENSG00000131018	SYNE1	6:152121780-152318344	10,742	−4.720139	6.59E-08	DOWN
ENST00000523924	ENSG00000066827	ZFAT	8:134477792-134713038	4,756	−4.861013	7.41E-08	DOWN
ENST00000512834	ENSG00000164306	PRIMPOL	4:184649725-184694952	2,174	−6.31489	4.52E-07	DOWN
ENST00000376135	ENSG00000138443	ABI2	2:203328459-203383296	568	−3.778507	1.42E-06	DOWN
ENST00000409694	ENSG00000131018	SYNE1	6:152121780-152311167	10,634	−5.08739	1.41E-06	DOWN
ENST00000371319	ENSG00000157216	SSBP3	1:54226639-54406008	1,578	−4.948243	1.76E-06	DOWN
ENST00000480283	ENSG00000085224	ATRX	X:77505713-77786216	10,455	−7.603071	2.22E-06	DOWN
ENST00000533655	ENSG00000165490	DDIAS	11:82901727-82934659	3,533	−3.847898	2.19E-06	DOWN

*Fold-change (FC) is presented as lncRNAs expression in women with vs. without polycystic ovary syndrome (PCOS). Up and Down are presented as the expression trend of mRNAs in women with vs. without polycystic ovary syndrome (PCOS)*.

### Gene Ontology and Kyoto Encyclopedia of Genes and Genomes Pathway Analyses

GO and KEGG were used to predict the functions of genes. The top 30 GO and KEGG enriched categories were determined (Figure 1D). The prominent GO enriched categories were associated with androgen and steroid hormone metabolism. The pathways closely linked to PCOS in the KEGG analysis were ovarian steroid production, lipid metabolism, immune signaling pathways, and the PI3K–Akt signaling pathway.

### Confirmation of LncRNA and mRNA Expression

**Figure 2:**
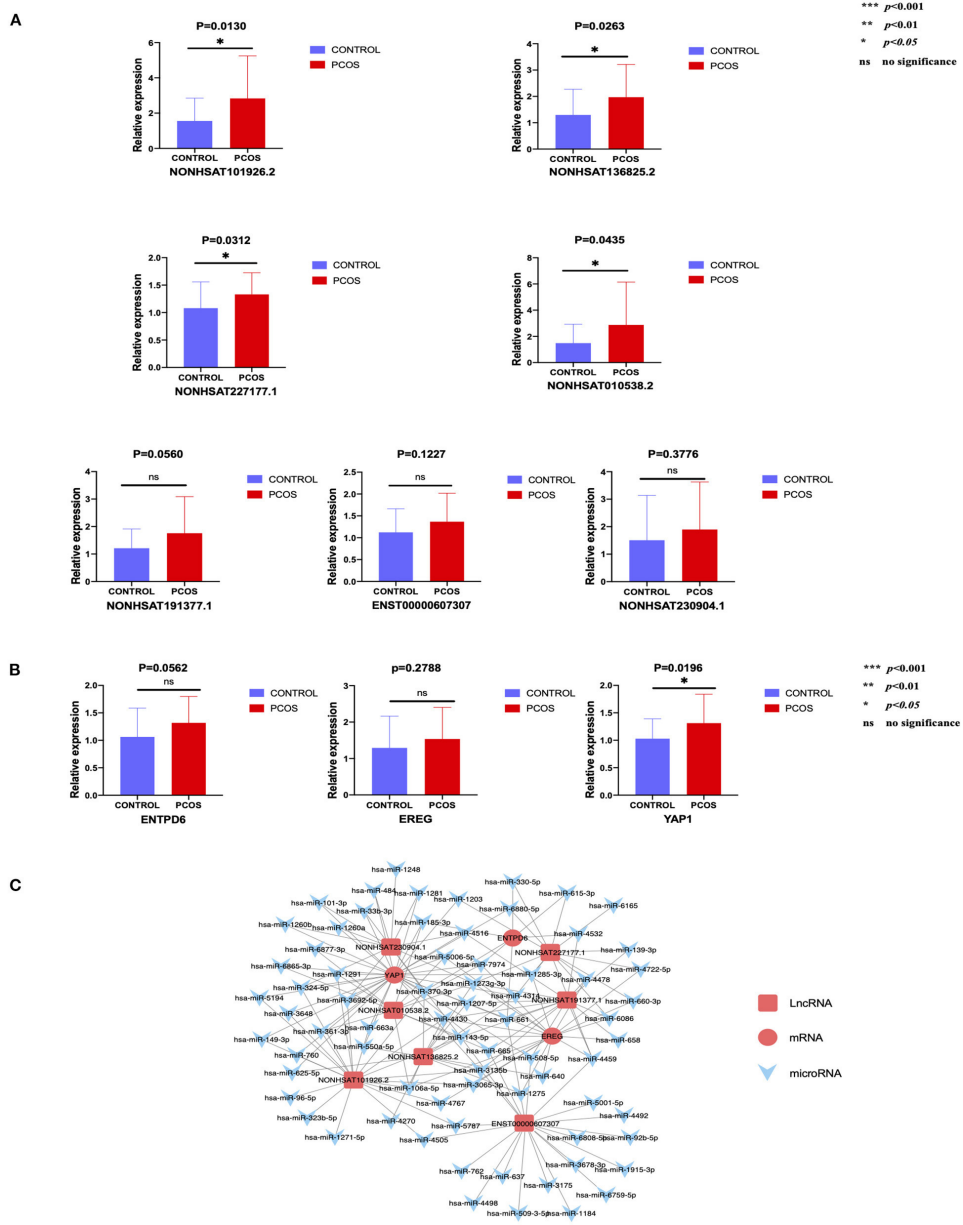
The expression level of lncRNAs and mRNAs conforming to the sequencing results and the predicted network regulatory relationship between them. **(A)** 7 lncRNAs that meet the trend of sequencing results. **(B)** 3 mRNAs that meet the trend of sequencing results. **(C)** Possible regulatory relationship between lncRNAs and mRNAs.

In order to conduct follow-up study, 34 lncRNAs and 10 mRNAs were randomly selected to verify their expression trend in PCOS group and control group by qRT-PCR. These lncRNAs and mRNAs were verified with 30 ovarian GC samples in each two groups. The screening criteria include *p* < 0.05, |fold-change| ≥2, sequence length <3,000bp and counts >10. After verification, it was found that the expression of 7 lncRNAs (NONHSAT101926.2, NONHSAT136825.2, NONHSAT227177.1, NONHSAT010538.2, NONHSAT191377.1, NONHSAT230904.1, ENST00000607307) and 3 mRNAs (YAP1, ENTPD6 and EREG) among the lncRNAs and mRNAs that have been screened were consistent with the sequencing results. The results in this paper showed that the expression trends of these lncRNAs and mRNAs were consistent with the sequencing results (Figure 2). The expression levels of 4 lncRNAs (NONHSAT101926.2, NONHSAT136825.2, NONHSAT227177.1, NONHSAT010538.2) among the 7 lncRNAs were consistent with the sequencing results with a significant difference between the two groups (*p* < 0.05). Meanwhile, the changes of the levels of the other 3 lncRNAs (NONHSAT191377.1, NONHSAT230904.1, ENST00000607307) lost statistical significance (*p* > 0.05). The expression levels of YAP1 among the 3 mRNAs were significant (*p* < 0.05), and the changes of the levels of the other 2 mRNAs (ENTPD6, EREG) did not reach statistical significance (*p* > 0.05) (Figure 2). The details of the fold changes and *p*-values of these lncRNAs and mRNAs as mentioned above are shown in Table 6.

**Table 6:** The log_2_fold-change (log_2_FC) and *p*-value of lncRNAs and mRNAs.

**lncRNAs/mRNAs**	**log_**2**_FC**	***p*** **-value**
NONHSAT101926.2	5.6813	7.23E-07
NONHSAT230904.1	3.7815	0.0102
NONHSAT136825.2	3.3254	0.0034
NONHSAT010538.2	1.5929	0.0009
NONHSAT227177.1	2.2648	0.0242
NONHSAT191377.1	3.1713	0.0241
ENST00000607307	1.4283	0.0114
ENTPD6	1.5899	0.0080
EREG	1.0764	0.0286
YAP1	2.1348	0.0089

### Construction of the LncRNA–MicroRNA-MRNA Interaction Network

According to our verification results, Cytoscape software was used to construct a lncRNA–microRNA-mRNA interaction network (Figure 2) to further speculate on the possible mutual regulatory relationship between lncRNA, microRNA and mRNA.

### Analysis of the Correlation Between LncRNAs and Clinical Parameters

**Figure 3:**
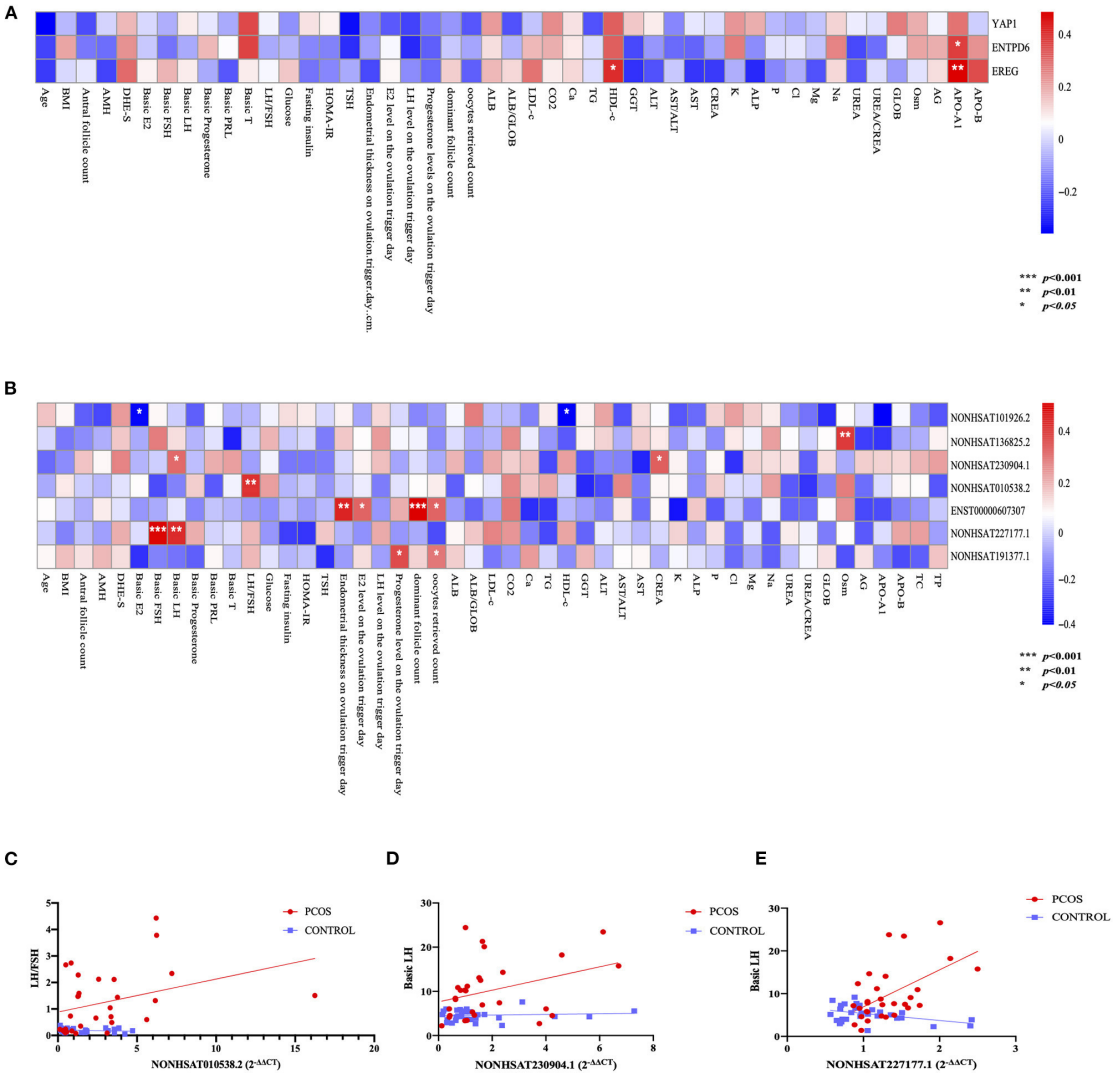
Correlation between polycystic ovary syndrome (PCOS) phenotypes and long non-coding RNAs (lncRNAs). **(A)** Binary correlation analysis between 3 mRNAs and PCOS phenotypes. **(B)** Binary correlation analysis between 7 lncRNAs and PCOS phenotypes. **(C)** Scatter diagram and linear correlation analysis of luteinizing hormone (LH)/follicle-stimulating hormone (FSH) ratio and NONHSAT010538.2 (*r* = 0.4905, *p* = 0.0059). **(D)** Scatter diagram and linear correlation analysis of basic luteinizing hormone (LH) level and NONHSAT230904.1 (*r* = 0.3813, *p* = 0.0412). **(E)** Scatter diagram and linear correlation analysis of basic luteinizing hormone (LH) level and NONHSAT227177.1 (*r* = 0.5046, *p* = 0.0045).

**Figure 4:**
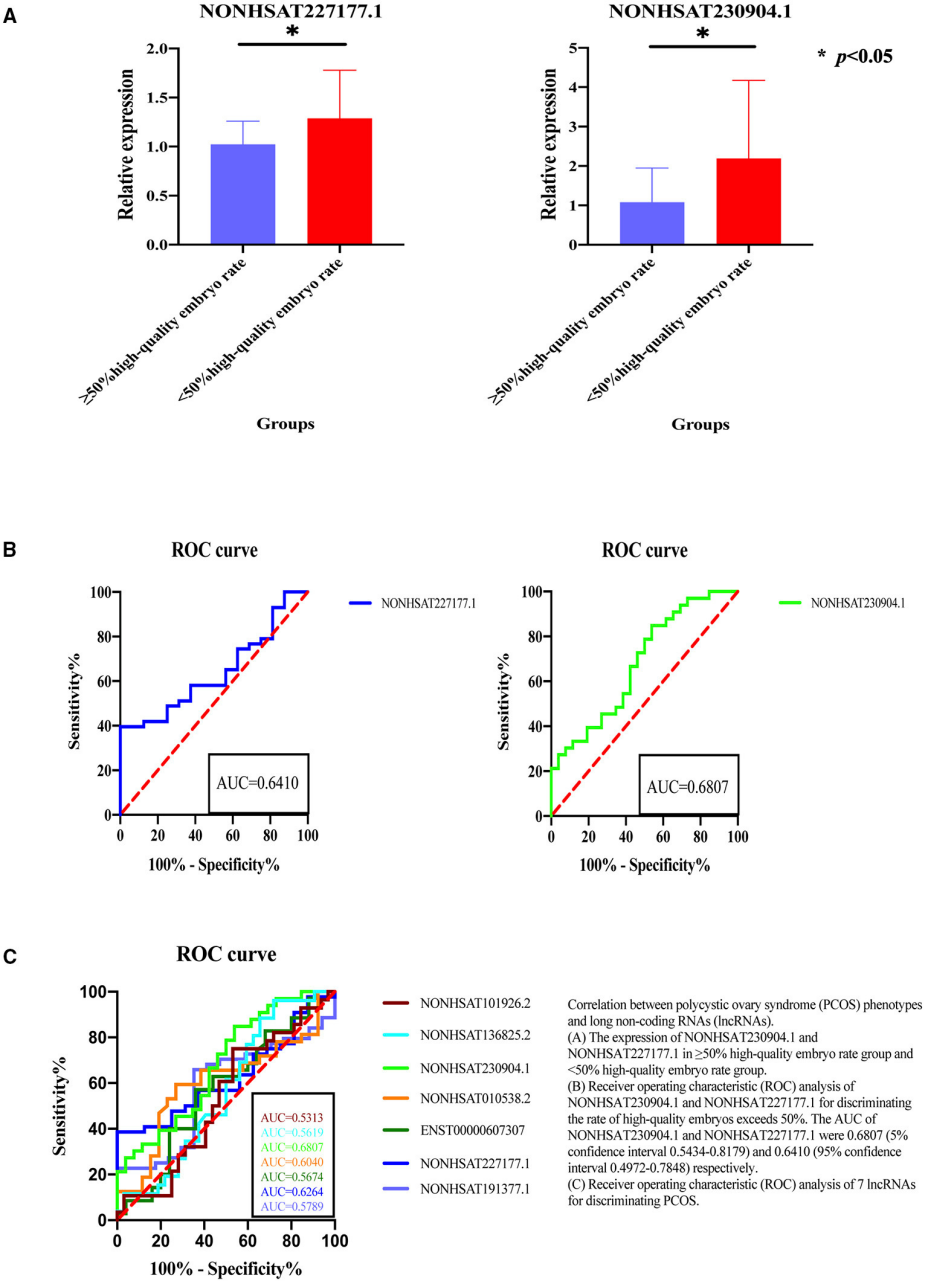
Correlation between polycystic ovary syndrome (PCOS) phenotypes and long non-coding RNAs (lncRNAs). **(A)** The expression of NONHSAT230904.1 and NONHSAT227177.1 in ≥50% high-quality embryo rate group and <50% high-quality embryo rate group. **(B)** Receiver operating characteristic (ROC) analysis of NONHSAT230904.1 and NONHSAT227177.1 for discriminating the rate of high-quality embryos exceeds 50%. The AUC of NONHSAT230904.1 and NONHSAT227177.1 were 0.6807 (5% confidence interval 0.5434-0.8179) and 0.6410 (95% confidence interval 0.4972–0.7848) respectively. **(C)** Receiver operating characteristic (ROC) analysis of 7 lncRNAs for discriminating PCOS. *p < 0.05.

Pearson's correlation analysis was performed between several PCOS phenotypes and the expression of mRNAs (YAP1, EREG, ENTPD6) and lncRNAs (NONHSAT101926.2, NONHSAT136825.2, NONHSAT227177.1, NONHSAT010538.2, NONHSAT191377.1, ENST00000607307, NONHSAT230904.1), which have been confirmed consistent with sequencing results. The results showed that EREG had positive relationship of high-density-lipoprotein c (HDL-c) and apolipoprotein A1 (ApoA1), ENTPD6 had positive relationship of APO A1 (Figure 3). On the other hand, significant negative relationships of plasma E_2_, HDL-c, and ApoA1 levels with NONHSAT101926.2 were noted. In contrast to the case in Controls, the plasma LH level was positively correlated with NONHSAT230904.1 and NONHSAT227177.1; such a correlation was also noted between plasma basic FSH and NONHSAT227177.1. The LH/FSH ratio was positively correlated with NONHSAT010538.2 expression. Additionally, the endometrial thickness on the day of trigger, plasma E_2_ level on the ovulation trigger day, number of dominant follicles and oocytes retrieved were all positively correlated with ENST00000607307. Plasma progesterone level on the ovulation day and number of oocytes retrieved were positively correlated with NONHSAT191377.1 (Figure 3). The ROC curve of all 7 lncRNAs demonstrated a better accuracy in distinguishing patients with PCOS from Controls (Figure 4; Table 7).

**Table 7:** Receiver operating characteristic (ROC) analysis of 7 lncRNAs to determine effectiveness in diagnosing PCOS.

**LncRNAs**	**Cut-off value**	**Sensitivity**	**Specificity**	**AUC (95% CI)**
NONHSAT101926.2	2.7447	0.467	0.833	0.6728 (0.5364–0.8092)
NONHSAT136825.2	1.8101	0.533	0.75	0.6744 (0.5368–0.8120)
NONHSAT230904.1	1.4875	0.448	0.767	0.5948 (0.4485–0.7411)
NONHSAT010538.2	0.7283	0.767	0.536	0.6536 (0.5117–0.7954)
ENST00000607307	1.209	0.7	0.7	0.6272 (0.4798–0.7746)
NONHSAT227177.1	0.9661	0.867	0.567	0.7244 (0.5928–0.8561)
NONHSAT191377.1	1.13	0.697	0.571	0.6055 (0.4631–0.7479)

According the quality of embryos standard: low-quality embryos have 2–7 blastomeres (fragmentation >20%) and high-quality embryos have 8 or more blastomeres (fragmentation ≤ 20%) (31), we divided the patients into two groups: the group with high-quality embryos exceeding 50% of the total number of embryos (≥50% high-quality embryo rate) and the group with high-quality embryos below 50% (<50% high-quality embryo rate). We analyzed the expression of lncRNAs between different embryo quality (rate of high-quality embryos exceeds 50% or not) and the results showed that the NONHSAT230904.1 and NONHSAT227177.1 expressed significantly different (Figure 4). Further, these two lncRNAs had a fine discrimination in embryo quality, with the AUC of were 0.6807 (95% CI, 0.5434–0.8179) and 0.6410 (95% CI 0.4972–0.7848), for NONHSAT230904.1 and NONHSAT227177.1, respectively (Figure 4).

## Discussion

High-throughput sequencing technology could provide a wealth of information about coding and non-coding RNAs that may be involved in PCOS. In this study, seven unfamiliar lncRNAs showed significant increases in PCOS patients. Functional enrichment of the lncRNAs and mRNAs differentially expressed in PCOS suggested their associations with androgen, steroid hormone metabolism, ovarian steroid production, lipid metabolism, immune signaling pathways, and the PI3K–Akt signaling pathway.

The mRNAs that were selected at random for screening, including EREG, YAP1, ENTPD6, were all upregulated in PCOS patients. So far, the research on PCOS for these three mRNAs has been covered, but there are limitations.

EREG can regulate the proliferation and apoptosis of GCs, and is positively correlated with the proliferation of ovarian GCs (32). Some studies have suggested that it is also related to insulin secretion (33). YAP1 participates in many signaling pathways that regulate organic morphology, including ovarian enlargement, which is a major manifestation of PCOS. YAP1 is highly expressed in the GCs of PCOS patients (34). Testosterone (T) and estradiol (E2) induce the hyperactivation of YAP1 to stimulate GC proliferation, which may participate in hyperandrogenism-induced oligo-ovulation (35). In addition, the overexpression of YAP can promote the proliferation of human pancreatic β cells and maintain their insulin secretion (36). Regarding ENTPD6, in research on obesity using genetic, functional, and computational follow-up analyses, Turcot et al. found that it may play a role in controlling energy intake and consumption, but further research on this is still needed (37). In addition, Rich et al. performed a genome-wide association study on the acute insulin response to glucose in Hispanic Americans, finding that ENTPD6 may be related to pancreatic β-cell response, the acute insulin response to glucose (AIRg) (38). According to the above results, EREG, YAP1 and ENTPD6 may be involved in the pathogenesis of PCOS, but the detailed pathogenic mechanisms remain unclear. Further experiments would be performed to demonstrate and explain the mechanism of action.

In our profiles, GO analysis indicated that the differentially expressed mRNAs are mainly related to androgen and steroid hormone metabolism. Meanwhile, the KEGG pathway analysis results indicated that these aberrantly expressed mRNAs were associated with ovarian steroid production, lipid metabolism, immune signaling pathways, and the PI3K–Akt signaling pathway, among others. In recent years, many studies have been performed on the involvement of lncRNAs in the pathogenesis of PCOS. Some sequencing results were also obtained showing that there were more significant differences in coding and non-coding RNAs between PCOS patients and Controls. Jiao et al. conducted a study on the characterization of lncRNA and mRNA profiles in follicular fluid from mature and immature ovarian follicles of women and PCOS patients. Their sequencing profiles showed that the lncRNAs with significant differences in expression were mainly linked to metabolism, nervous system, and olfactory transduction pathways (6). Moreover, deep sequencing of mRNA and lncRNA profiles in a letrozole-induced PCOS rat model showed that the differentially expressed mRNAs were associated with biological adhesion, reproduction, and metabolic processes, and they were also enriched in several specific signaling pathways, including insulin resistance, steroid hormone biosynthesis, PPAR signaling pathway, cell adhesion molecules, autoimmune thyroid disease, and AMPK signaling pathway (39). Furthermore, a pilot study found significant or borderline significant increases in several parameters reflecting intestinal barrier dysfunction and inflammation in PCOS patients, suggesting that PCOS may be related to metabolic disorders (40). This summary from previous works shows that our sequencing profiles exhibit some differences from others' results. The reason for these discrepancies may be that we selected different sample tissues.

In recent years, many studies focused on the relationship between lncRNAs and PCOS. Li et al. found that the lncRNA SRLR participated in PCOS by upregulating IL-6 and promoting apoptosis of human granulosa-like tumor cells (KGN) (41). Geng et al. showed that the lnc-MAP3K13-7:1 inhibited the ovarian GCs proliferation in PCOS *via* DNMT1 inhibiting CDKN1A/p21 expression (42). Some PCOS animal model studies also provided evidences that lncRNAs might be used as therapeutic targets for PCOS. Jiang et al. observed a down-regulated of lncRNA HOTAIR could alleviates PCOS (43). And Yang et al. showed that silencing the lncRNA UCA1 could inhibit the development of PCOS *via* regulating PI3K-AKT signaling pathway (44). Moreover, downregulating lncRNA NEAT1 could also inhibit apoptosis and improve cell proliferation of ovarian GCs *via* microRNA-381/IGF1 axis, which associated with improved pathological process of PCOS (45). And our results exhibited that the expression of lncRNA was also significantly correlated with abnormal hormone, plasma metabolites level and embryo quality: NONHSAT101926.2 was mainly related to lipid metabolism, NONHSAT227177.1 and NONHSAT230904.1 were mainly related to plasma steroid hormones, NONHSAT010538.2 was positively associated with the LH/FSH ratio, while ENST00000607307 and NONHSAT191377.1 were related to plasma steroid hormones and follicle development. Moreover, we also observed high expression of NONHSAT230904.1 and NONHSAT227177.1 were associated with fewer high-quality embryos, which provided evidence that they could be related to embryo quality. Therefore, we speculate that these 7 lncRNAs and 3 mRNAs mentioned above were likely to be invovled in the pathogenesis of PCOS and might become a new molecular therapeutic targets for PCOS.

However, there are several limitations in this study. We only introduced our high-throughput sequencing profiles and they need to be confirmed on a larger cohort. Likewise, we have analyzed the relationships between lncRNAs, mRNAs and PCOS clinical parameters, but further in-depth research is needed to elucidate the exact mechanisms linking these RNAs to PCOS.

## Conclusion

Our findings expanded the knowledge that abnormal expression of lncRNAs and mRNAs mentioned in present study in ovarian granulosa cells were associated with PCOS, which also could be reflected in abnormal hormone, plasma biomedical indicators and embryo quality. And these preliminary findings might enlight some new directions for understanding PCOS.

## Data Availability Statement

The original contributions presented in the study are publicly available. This data can be found here: https://www.ncbi.nlm.nih.gov/geo/query/acc.cgi?acc=GSE173160.

## Ethics Statement

The studies involving human participants were reviewed and approved by the Ethics Committee of the Reproductive Hospital Affiliated to Shandong University. The patients/participants provided their written informed consent to participate in this study.

## Author Contributions

YZ and YS conceived and designed this study. RW, WC, and XY collected clinical data. YZ, YB, PL, LF, YW, and SZ contributed to statistical analysis. YZ drafted the manuscript. YZ, YB, and YS participated in the discussion and critically revised the article. All authors contributed to the article and approved the submitted version.

## Funding

This study was supported by National Key R&D Program of China (2018YFC1003202 and 2017YFC1001004) and Taishan scholar project special funds (No. ts201712103).

## Conflict of Interest

The authors declare that the research was conducted in the absence of any commercial or financial relationships that could be construed as a potential conflict of interest.

## Publisher's Note

All claims expressed in this article are solely those of the authors and do not necessarily represent those of their affiliated organizations, or those of the publisher, the editors and the reviewers. Any product that may be evaluated in this article, or claim that may be made by its manufacturer, is not guaranteed or endorsed by the publisher.
